# Non-volatile artificial synapse based on a vortex nano-oscillator

**DOI:** 10.1038/s41598-021-95569-4

**Published:** 2021-08-09

**Authors:** Leandro Martins, Alex S. Jenkins, Lara San Emeterio Alvarez, Jérôme Borme, Tim Böhnert, João Ventura, Paulo P. Freitas, Ricardo Ferreira

**Affiliations:** 1 INL, Avenida Mestre José Veiga, s/n, 4715-330 Braga, Portugal; 2 IFIMUP-IN, Rua do Campo Alegre, 678, 4169-007 Porto, Portugal

**Keywords:** Materials science, Nanoscience and technology, Physics

## Abstract

In this work, a new mechanism to combine a non-volatile behaviour with the spin diode detection of a vortex-based spin torque nano-oscillator (STVO) is presented. Experimentally, it is observed that the spin diode response of the oscillator depends on the vortex chirality. Consequently, fixing the frequency of the incoming signal and switching the vortex chirality results in a different rectified voltage. In this way, the chirality can be deterministically controlled via the application of electrical signals injected locally in the device, resulting in a non-volatile control of the output voltage for a given input frequency. Micromagnetic simulations corroborate the experimental results and show the main contribution of the Oersted field created by the input RF current density in defining two distinct spin diode detections for different chiralities. By using two non-identical STVOs, we show how these devices can be used as programmable non-volatile synapses in artificial neural networks.

## Introduction

In the past decades, artificial neural networks (ANNs) have been extensively studied with excellent results in many domains of science, being image^1^ and speech^2^ recognition notable examples that have been extended to important areas, such as precision medicine^3^. These networks are made up of several layers of artificial neurons interconnected via artificial synaptic connections which require a programmable weighting mechanism between outputs and inputs to enable the artificial network to learn. ANNs implemented in software have been successful, but power efficient and scalable hardware implementations of such systems are lagging behind. To date, various technologies have been proposed to implement neuromorphic computing, namely optical^4^, semiconductor^5,6^, bioelectronic^7^, carbon-based^8^, superconducting^9^ and memristive^10^ devices.

In recent years, spintronic devices have also been proposed for the hardware implementation of neuromorphic computing systems^11–13^. Spin torque nano-oscillators (STNOs) are among the most studied spintronic devices for use in ANNs^14–21^. An STNO consists of a spin valve^22,23^ or, more often, an MgO-based magnetic tunnel junction (MTJ)^24,25^, combining a resistive output which depends on the magnetization state of a ferromagnetic layer with rich magnetization dynamics that can be manipulated by spin-polarized currents^26,27^. These RF oscillators show a high degree of versatility since, depending on the input signal, they can work as nano-scale RF sources^28,29^ as well as RF detectors^30–33^.

Significant advances have been made recently^14–20^, with reports showing STNO neural capabilities in reservoir computing systems^16–18^, where the transient dynamic state of the oscillator (i.e. short-term memory) is used to compute time-varying input signals. Non-trivial tasks such as vowel recognition were achieved in a synchronization-based computing system^19^, where the recognition is performed based on the synchronization state between the input signal and a chain of four STNOs representing artificial neurons. More recently, STNOs have also been proposed to work as synapses in ANNs where, based on the spin diode effect, the synaptic weight depends on the frequency difference between the input RF signal and the resonator^20,21^. In this work, we propose the possibility of adding non-volatility to an STNO working as an artificial synapse, which in turn enables learning in an ANN using such devices. Specifically, we show that the detection (i.e. synaptic weight) of a vortex-based spin torque nano-oscillator (STVO) by spin diode effect depends on the vortex chirality and can be persistently changed after fabrication by means of a proper initialization via electrical signals applied selectively to each device alone.

## Experimental details

**Figure 1:**
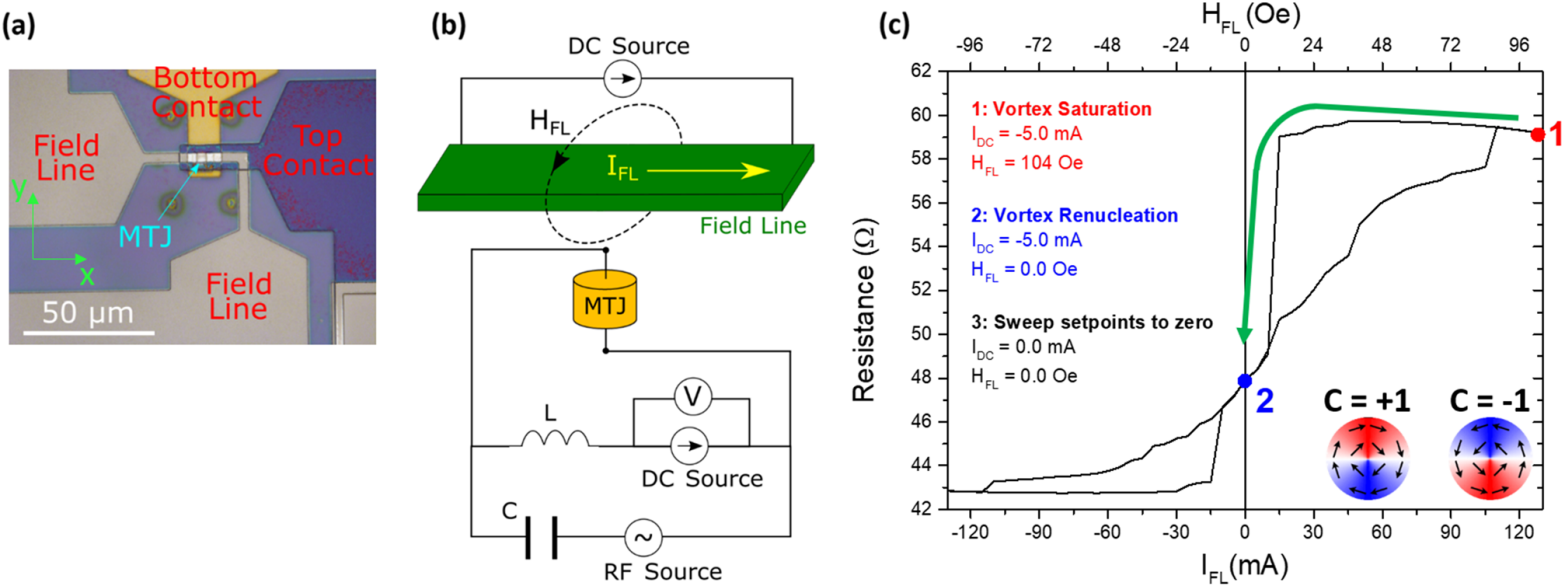
(**a**) Top view optical image of the devices after fabrication with an integrated local field line fabricated on top of the MTJ nanopillar. (**b**) Illustration of the experimental setup used for the initialization and spin diode measurements. (**c**) Two terminal R-H loop for an MTJ with a diameter of 1.0 µm, explaining the 3-step initialization process. The insets show how each chirality is associated with a different vortex configuration of the in-plane magnetic moments of the free layer, as seen from the bottom of the MTJ.

This work is based on circular nanopillars with diameters of 0.9 μm and 1.0 μm, patterned from a 6.0 IrMn/2.0 CoFe_30_/0.7 Ru/2.6 CoFe_40_B_20_/0.8 MgO/2.0 CoFe_40_B_20_/0.2 Ta/7.0 NiFe MTJ stack (thickness in nanometers). The MgO barrier thickness results in a resistance-area product of 8.0 Ω µm^2^ (resistance of the parallel configuration of the MTJ considered) and the 2.0 CoFe_40_B_20_/0.2 Ta/7.0 NiFe free layer provides a remanent vortex state^34^. A field line antenna with a width of 3 µm is vertically integrated with the nanopillar, at a distance of approximately 600 nm on top of the free layer (Fig. 1a). The field line antenna allows the application of a local in-plane magnetic field (Fig. 1b) along the reference direction (yy axis in Fig. 1a) set by the synthetic antiferromagnetic structure (SAF), which is defined by a 1 Tesla magnetic field applied during the cool down of an annealing step performed at a temperature of 330 °C with a duration of 2 h. In this geometry, the field line is capable of producing a local magnetic field large enough to saturate the free layer, so Fig. 1c shows how the field line in conjunction with the local bias current applied to the MTJ can be used to set the vortex chirality. Here, a positive DC bias current corresponds to electrons traveling from the free to the reference layer and a positive chirality (C =  + 1) corresponds to a clockwise rotation of the free layer magnetization as seen from the bottom (see inset of Fig. 1c). The protocol used to deterministically set the vortex chirality can be described as a three-step initialization of the vortex. In the first step, a DC bias current (I_DC_) is applied directly through the MTJ, together with a field line current producing a local magnetic field large enough to saturate the free layer (point 1). In the second step, the magnetic field is reduced down to a value that results in the renucleation of the vortex (point 2). In this example, the magnetic field was reduced down to zero (but a range of other values is possible in accordance to the specific R-H loop of the device) and the bias current amplitude is set to 5.0 mA (which results in a current density of 6.4 × 10^9^ A/m^2^). At this point, the chirality of the vortex is defined by the direction of the Oersted field associated with the DC bias current^35^. A positive bias I_DC_ results in the nucleation of a vortex with a positive chirality, while a negative bias I_DC_ results in the nucleation of a vortex with a negative chirality. In the third step, the magnetic field and bias I_DC_ are set to zero. After this process is complete, the vortex chirality is defined and remains stable. This process of setting the vortex chirality is completely deterministic, so that switching the vortex to the opposite chirality is achieved simply by repeating the process with an initialization bias current applied in the opposite direction. Once the vortex is initialized with a specific chirality, a spin diode measurement is performed using a bias tee to apply an RF signal with a sweeping frequency directly to the MTJ and measuring as output the rectified voltage across the MTJ nanopillar, as shown in Fig. 1b.

## Results

**Figure 2:**
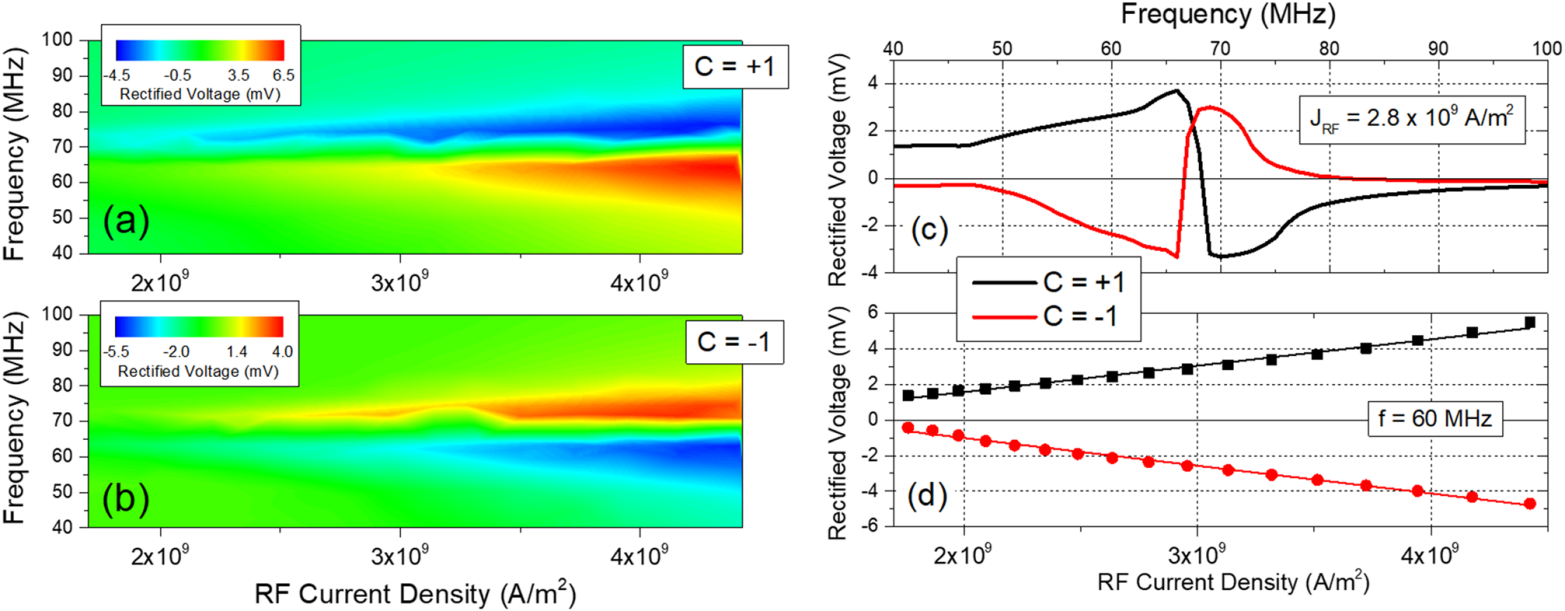
Spin diode measurements presented as a function of the input RF current density for (**a**) positive and (**b**) negative chiralities. (**c**) Spin diode measurement obtained for an input RF current density of 2.8 × 10^9^ A/m^2^. (**d**) Rectified voltage measured at a fixed frequency of 60 MHz and presented as a function of the input RF current density.

Figure 2a,b show a spin diode measurement obtained from an MTJ with a diameter of 1.0 µm, as a function of the input RF current density (J_RF_) at zero external magnetic field. There is an obvious dependence of the spin diode output on the chirality of the vortex. The difference between the two chiralities is made evident in Fig. 2c, obtained by measuring the spin diode voltage output as a function of the RF current frequency for a fixed RF amplitude, corresponding to a current density of 2.8 × 10^9^ A/m^2^ across the MTJ. The output obtained for each chirality shows a degree of symmetry with respect to the central frequency of detection. In Fig. 2d, the frequency of the RF signal is fixed at 60 MHz (a point close to the central frequency) and the RF signal amplitude is swept, resulting in a rectified voltage that is symmetric with respect to each of the two chiralities across the whole range. It is also worth noting that, for both chiralities, the spin diode voltage output is proportional to the RF signal amplitude, indicating that the nonlinear characteristics of the detector^36^ have a small effect with this specific geometry and current density range. In this way, the STVO can function as an artificial synapse whose weight can be persistently changed in a binary and non-volatile scheme, so that the rectified voltage measured for positive (V^+^) and negative (V^−^) chiralities is given by^21^

1 $${V}^{+}={W}^{+} \cdot {J}_{RF}$$

or

2 $${V}^{-}={W}^{-} \cdot {J}_{RF},$$

where W^+^ and W^−^ are the synaptic weights (i.e. slope of each linear fit in Fig. 2d) for each chirality. Similar to an STT-MRAM^37^ , where a large current density is used to set MTJs between two possible states that are read at low current densities, the initialization step allows to switch the chirality of the vortex, which in turn changes the spin diode output at fixed values of input RF signal amplitude and frequency. The synaptic weight is proportional to the output voltage measured for a given J_RF_. In turn, the output voltage depends on the amplitude of the vortex core oscillation during resonance, in a way that, by TMR effect, a larger orbit of the core leads to a larger output voltage. Thus, to maximize the synaptic weights, it is required to maximize the radius of the vortex orbit. That radius depends in a complex way on a large number of material properties^31^ but, as an example, an obvious way to do it is to optimize the spin torque efficiency and/or decrease the Gilbert damping of the free layer^38^.

To have a physical insight of the experimental results, micromagnetic simulations were performed using the GPU-based software MuMax3^39^. The simulations comprised a circular nanodisk with a diameter of 1.0 µm and a thickness of 9.0 nm. The reference layer is set with an in-plane magnetization along the yy axis of the simulation plane. Based on experimental VSM data taken from NiFe films and standard values, the physical parameters were chosen as: saturation magnetization ${\mathrm{M}}_{\mathrm{S}}=680\times {10}^{3} \;\; \mathrm{ A}/\mathrm{m}$, uniaxial anisotropy constant along the yy axis ${\mathrm{K}}_{\mathrm{u}}=340 \;\; \mathrm{ J}/\text{m}^{3}$, Gilbert damping $\alpha =0.01$ and exchange stiffness $\mathrm{A}=1.3\times {10}^{-11}\;\;\mathrm{ J}/\mathrm{m}$. An RF current density was applied in the direction perpendicular to the nanodisk plane, being its radial Oersted magnetic field also considered, with a spin-polarization $\mathrm{P}=0.4$ and a secondary spin-torque term ${\varepsilon }{^{\prime}}=0.4$. In this way, the simulated gyrotropic motion is considered to be driven, not only by the spin-transfer torque (STT)^40^ that naturally arises from the spin-polarized current, but also by a realistic Oersted field torque (OFT) coming from the RF current. The initial magnetization configuration for the micromagnetic simulations was set as a vortex with the two possible chiralities at a positive polarity. According to the simulations, the polarity switching does not change the spin diode results (see the supplementary information).

**Figure 3:**
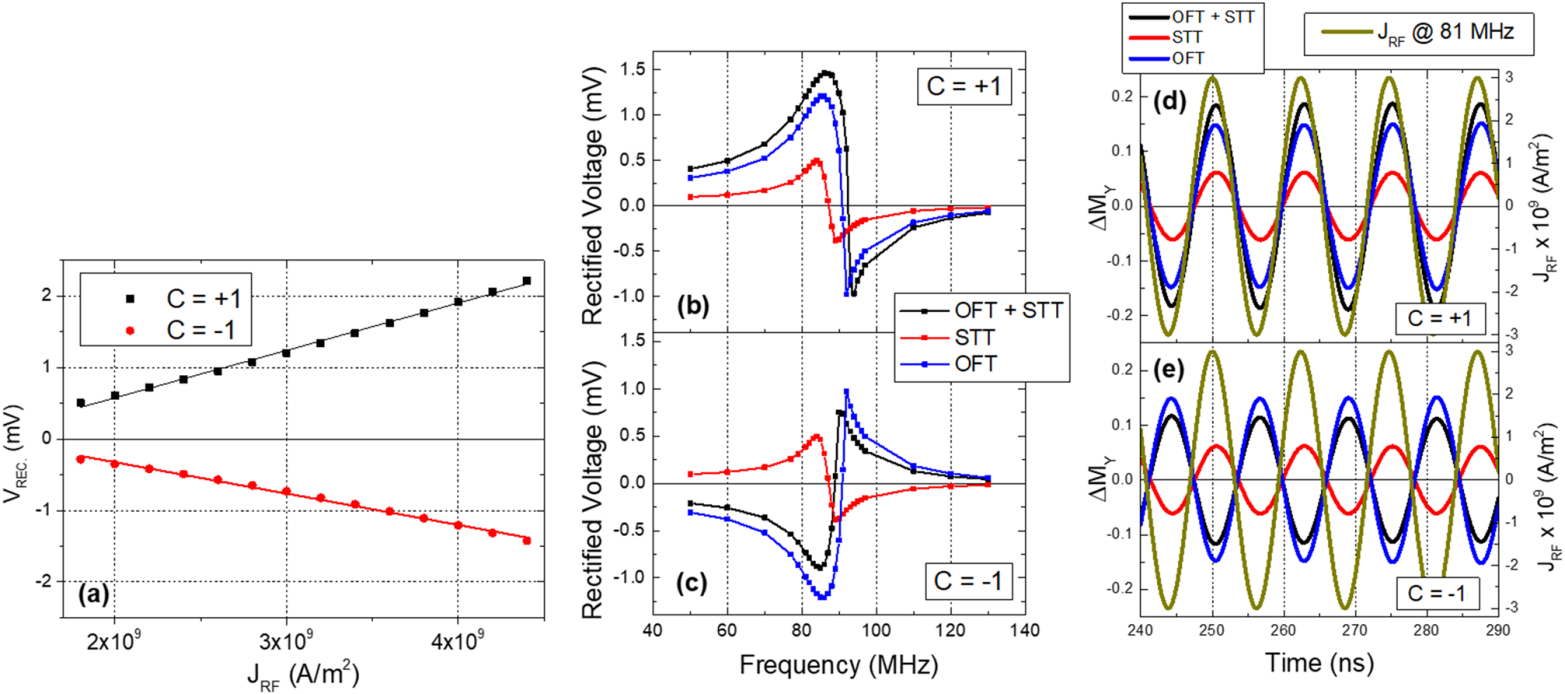
(**a**) Simulated rectified voltage calculated as a function of the input RF current density for the two different vortex chiralities at a fixed frequency of 81 MHz. An in-plane magnetic field of 39.8 Oe is applied in the yy direction. The simulated spin diode measurements for (**b**) positive and (**c**) negative chiralities are presented for different excitation mechanisms: spin-transfer torque (STT), Oersted field torque (OFT) and where both mechanisms are considered (OFT + STT). The results were obtained for an RF current density of 3.0 × 10^9^ A/m^2^. (**d**,**e**) Simulated ΔM_Y_ time traces obtained for both chiralities. Similar to (**b**) and (**c**), different excitation mechanisms are considered. The results were obtained for an RF current density of 3.0 × 10^9^ A/m^2^ at a frequency of 81 MHz whose time traces are also presented.

Figure 3a shows the amplitude of the simulated spin diode results, calculated as a function of the RF current density for both chiralities. The results are calculated at a fixed frequency of 81 MHz. The effective field (i.e. sum of all the internal and external magnetic fields) acting on the free layer^41,42^ leads to a displacement of the vortex core position from the geometric center of the pillar, which needs to be considered (i.e. in-plane magnetic field along the yy direction) to properly simulate the experimental conditions and was determined to be ${\mathrm{H}}_{\mathrm{eff}}=39.8\pm 0.3 (\mathrm{Oe})$ (see the supplementary information). The simulated nanodisk does not give a complete description of the real MTJ nanopillar, so that the frequency discrepancy between experimental and simulated data is expected. The rectified voltage (V_REC_) is estimated based on experimental data, so that

3 $${V}_{REC}=\frac{{R}_{AP}-{R}_{P}}{2}\cdot S\cdot \frac{1}{T}{\int }_{0}^{T}{\Delta M}_{Y}\left(t\right)\cdot {J}_{RF}\left(t\right) dt,$$

where R_P_ (R_AP_) is the resistance of the MTJ in the parallel (antiparallel) configuration, S the area of vortex (S = πd^2^/4, being d = 1.0 µm the diameter of the nanodisk) and ΔM_Y_ the variation in time of the normalized magnetization in the yy direction. To estimate V_REC_, values of R_P_ and R_AP_ are measured with a bias DC current of 0.1 mA from the MTJ device whose spin diode measurement is presented in Fig. 2 (see supplementary information). For each frequency value, the simulations are performed for a time period T = 300 ns. Similar to the experimental results, the voltage presents different signs, as a result of the two different lineshapes given by different chiralities (see OFT + STT curve from Fig. 3b,c). In order to study the role of the OFT in the gyrotropic motion, Fig. 3b,c also show the simulated spin diode detection when only one excitation mechanism is considered at a time: a pure STT-driven oscillation (red data) and a pure OFT-driven oscillation (blue data). For an STT-driven oscillation, the spin diode response is the same for both chiralities. On the other hand, the OFT-driven output shows two symmetric lineshapes which depend on the vortex chirality. As described in Eq. (3), V_REC_ is calculated according to the time-averaged phase difference between ΔM_Y_ and J_RF_. The OFT-driven oscillation leads to two perfectly symmetric lineshapes, meaning that a chirality switching results in a 180° phase shift of the OFT-driven ΔM_Y_ oscillation.

To see how the combination of the STT and OFT creates distinct spin diode lineshapes for different chiralities, Fig. 3d,e show time traces of the simulated gyrotropic motion for different oscillation-driven mechanisms. Again, the frequency of J_RF_ is 81 MHz, near the central frequency of detection and the results are presented for the two chiralities. The time-averaged phase difference ψ between ΔM_Y_ and J_RF_ can be defined as

4 $$\psi =\frac{1}{T}{\int }_{0}^{T}[ {\varphi }_{{\Delta M}_{Y}}\left(t\right)-{\varphi }_{{J}_{RF}}\left(t\right) ]dt$$

being ${\mathrm{\varphi }}_{{\Delta \mathrm{M}}_{\mathrm{Y}}}$ the phase of the ΔM_Y_ oscillation and ${\mathrm{\varphi }}_{{\mathrm{J}}_{\mathrm{RF}}}$ the phase of the RF current density. For C =  + 1, the STT and OFT oscillations are in-phase, so that their combination results in a larger gyrotropic orbit with ψ = − 15° which, according to Eq. (3), leads to a positive voltage of 1.2 mV (Fig. 3b). For C = − 1, the STT oscillation is unchanged, but due to the 180° shift of the OFT-driven oscillation, now they are out-of-phase, which damps the oscillation to a smaller orbit with ψ = 163°, leading to a negative voltage of − 0.7 mV (Fig. 3c). These results clearly demonstrate the major role of the Oersted field in defining the spin diode output voltage.

**Figure 4:**
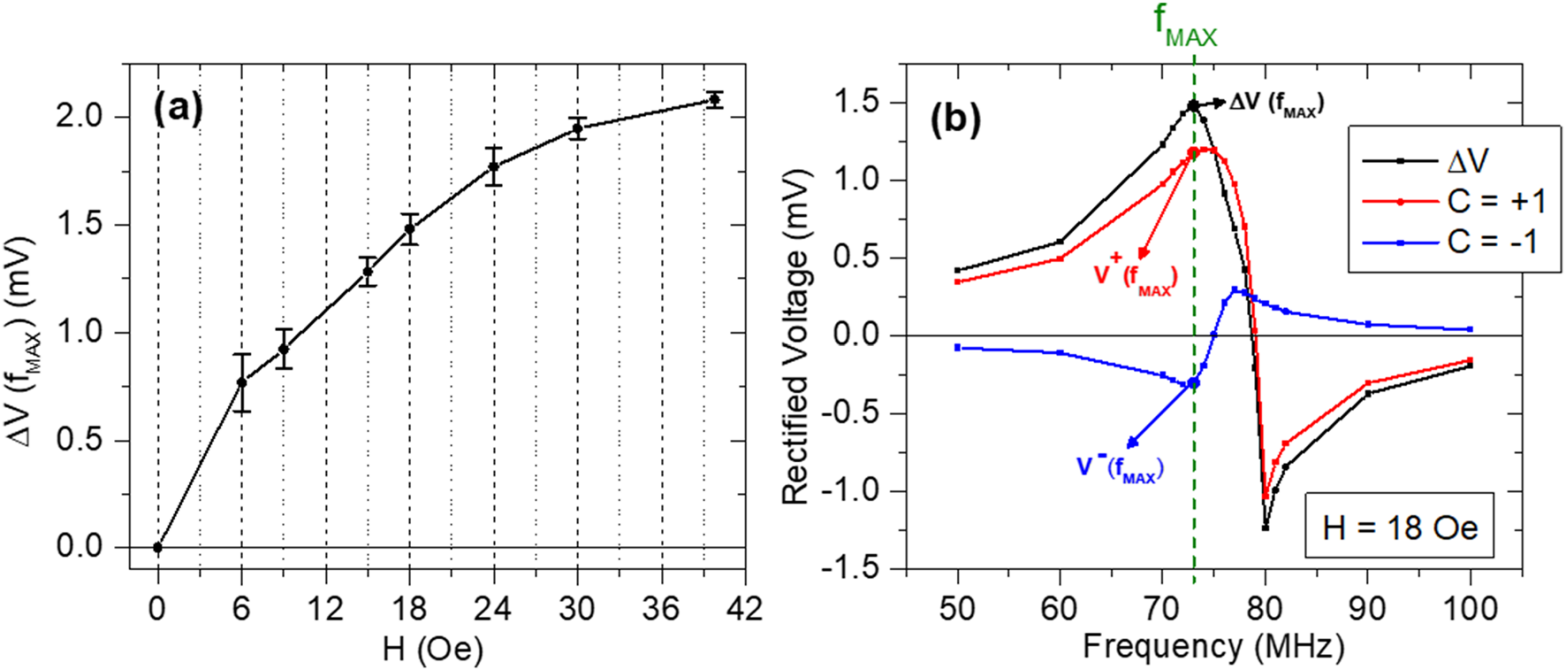
(**a**) Difference of the output voltage (ΔV) calculated for C =  + 1 and C = − 1. For each setpoint field, ΔV is calculated at the frequency f_MAX_ that maximizes the difference. (**b**) Example of the ΔV calculation for a setpoint field of 18.0 Oe.

It should be emphasized the importance of having a displacement of the vortex core at the remanent state. In fact, if the core is at the center, the spin diode output for C =  + 1 and C = − 1 should be the same (see the supplementary information). The Oersted field creates magnetic field lines whose center is also the central point of the pillar. When the core is at the center of the pillar, the vortex in-plane magnetization will be collinear with the Oersted field lines, so no oscillation of the vortex core is expected^33^. If there is no OFT-driven oscillation, the STT would be the only mechanism responsible for the gyrotropic motion, so that the M_Y_ oscillation would be the same for both chiralities. The role of the in-plane field acting on the free layer is clarified by simulated results shown in Fig. 4. For each value of the external magnetic field, the spin diode output is computed for the two chiralities at a fixed RF current density, as shown in Fig. 4b for the particular case of a setpoint field of 18.0 Oe. The interest relies on the frequency f_MAX_ that maximizes the difference ΔV(f_MAX_) = V^+^(f_MAX_) – V^−^(f_MAX_), where V^+^(f_MAX_) and V^−^(f_MAX_) are the output voltages calculated at f_MAX_ for C =  + 1 and C = − 1, respectively. Out of these results, ΔV(f_MAX_) is calculated for each value of the effective field acting on the free layer (Fig. 4a). As expected, at zero field, there is no distinction between the spin diode of different chiralities, so that ΔV = 0. The applied field allows to increase the vortex core displacement, which in turn increases the OFT contribution to the overall gyrotropic oscillation that breaks the symmetry of the resonance phenomenon (see supplementary information). Considering that the synaptic weight is proportional to the measured output voltage, these results suggest that the difference between W^+^ and W^−^ (i.e. non-volatile effect) can be optimized by promoting the maximum core displacement, which should only be limited to avoid a possible vortex core expulsion during the resonance process^31,43^, due to the proximity of the core with the pillar boundary.

**Figure 5:**
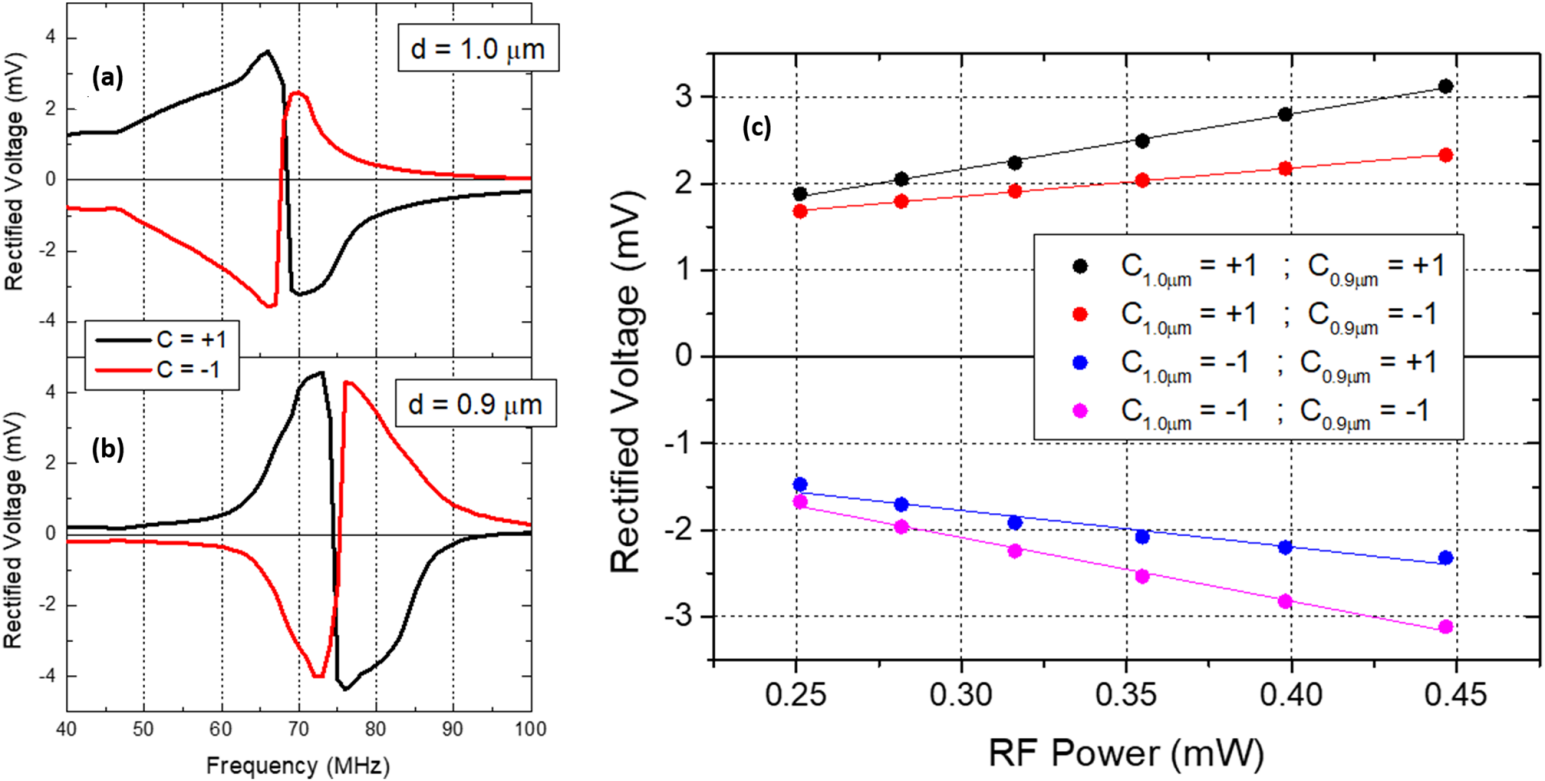
Spin diode measurements obtained for an STVO with a diameter of (**a**) 1.0 µm and (**b**) 0.9 µm, for an RF input power of 0.398 mW. The four possible programmable sums of the output voltages are presented in (**c**) as a function of the input RF power for a frequency of 60 MHz.

Once a non-volatile control of the synaptic weight is achieved for a single STVO, this control mechanism can be extended to a set of STVOs with different geometries and dynamic properties. Figure 5a,b demonstrates this possibility for a pair of circular STVOs with different diameters, where the spin diode outputs are presented for each pillar individually. Due to its lower dimensions, the MTJ with a diameter of 0.9 µm presents a larger resonance frequency than the MTJ with a diameter of 1.0 µm^43,44^, meaning that it is straightforward to program a fixed input frequency, so that the rectified voltage measured by each pillar is different. Figure 5c shows four possible spin diode outputs calculated as the sum of the output voltage measured individually for each pillar when subjected to a variable amplitude RF signal injected with a fixed input frequency of 60 MHz. Considering that each pillar has two possible synaptic weights (i.e. two possible chirality configurations), the system ends up with four possible programmable outputs (N non-identical STVOs lead to 2^N^ programmable outputs) that may vary depending on the frequency and power of the input signal. In addition to fabricate MTJ pillars with different diameters, there are other possible ways to obtain non-identical detectors. An alternative could be to change the thickness of the free layer by deposition of a free layer wedge during the nanofabrication process (i.e. changing the vortex aspect ratio^34^). This step would lead not only to a smooth variation of the resonance frequency over the wedge, but also to a smaller variation of the resistance between non-identical STVOs, which would be essentially dependent on the sample-to-sample variability. The resistance variability between STVOs should be minimized to make sure that, for the same input RF power, all the STVOs receive approximately the same RF current density.

## Conclusion

In conclusion, it is shown that a vortex-based spin torque nano-oscillator (STVO) working as a rectifier can be used as a binary non-volatile artificial synapse, enabling learning to be performed in ANNs making use of these devices. The synaptic weight can be persistently changed by switching the chirality of the vortex induced in the free layer. Micromagnetic simulations show that the Oersted field created by the RF current density is the main responsible for the symmetry breaking. It is also proposed to scale up the programming outputs by using two STVOs with different resonance frequencies, in this way opening new perspectives to a full integration of spintronic devices in neuromorphic hardware.

## Supplementary Information

Supplementary Information.
