# Supplementary material for: Non-volatile artificial synapse based on a vortex nano-oscillator
Source: Sci Rep. 2021 Aug 9;11:16094. doi: 10.1038/s41598-021-95569-4 (PMC8352962; doi:10.1038/s41598-021-95569-4)
Supplement: Supplementary file 1 — Supplementary Information. [file 41598_2021_95569_MOESM1_ESM.docx]

**Non-volatile artificial synapse based on a vortex nano-oscillator**

Leandro Martins*^1,2^, Alex S. Jenkins^1^, Lara San Emeterio Alvarez^1^, Tim Böhnert^1^, Jérôme Borme^1^, João Ventura^2^, Paulo P. Freitas^1^ and Ricardo Ferreira^1^

^1^ INL, Avenida Mestre José Veiga, s/n, 4715-330 Braga, Portugal

^2^ IFIMUP-IN, Rua do Campo Alegre, 678, 4169-007 Porto, Portugal

* leandro.martins@inl.int

**Supplementary information**

Figure S1 shows the R-H loop of the STVO whose results are presented in Fig. 2 of the main text. The data is used to calculate the effective field (H_eff_) applied to the free layer, which is the in-plane magnetic field applied in the simulations. The effective field leads to an offset of the vortex core from the center of the nanopillar in the remanent state. Moreover, when the core is at the center of the pillar, the resistance of the MTJ (R_C_) is given by

$$R_{C}=\frac{R_{AP}-R_{P}}{2}$$

where R_P_ (R_AP_) is the MTJ resistance in the parallel (antiparallel) configuration. The complete R-H loop shown in Fig. S1 allows to calculate averaged values of R_P_ = 38.0 Ω and R_AP_ = 50.2 Ω. The partial R-H loop from Fig. S1 is used to calculate the field at which the core is located at the center of the pillar. Before measuring the partial R-H loop, the vortex is initialized according to the INIT process illustrated in Fig. 1(c) of the main text. Then, the partial loop is measured in a field range not enough to annihilate the vortex (there is an absence of coercivity). Finally, based on the partial loop, the effective field H_eff_ = 39.8 ± 0.3 (Oe) corresponds to the setpoint at which R_MTJ_ = R_C_ = 44.1 Ω, being R_MTJ_ the resistance of the MTJ pillar.


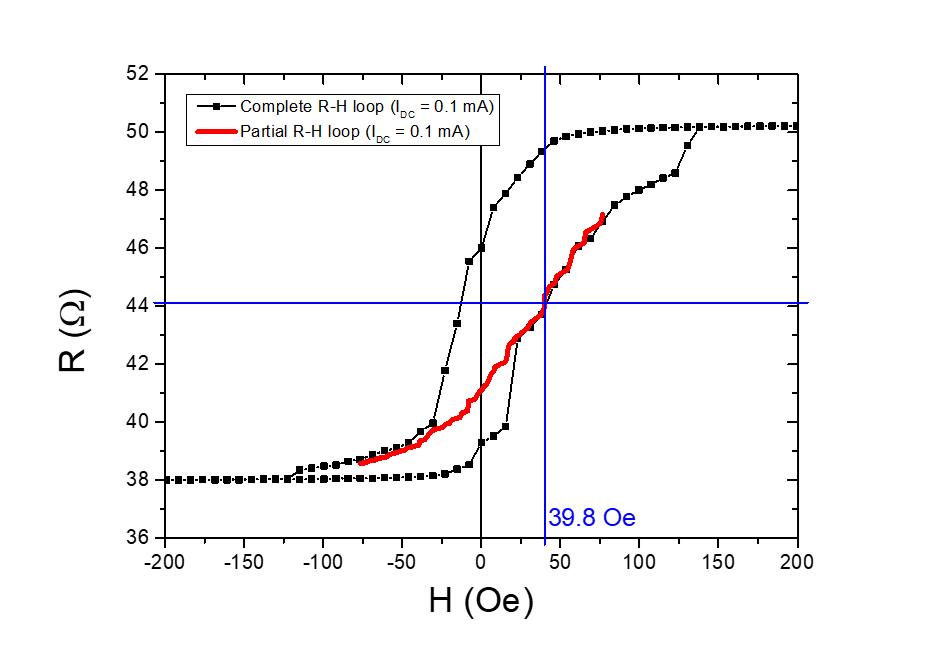


Figure S1: Complete and partial R-H loops obtained for a DC current of 0.1 mA. The results refer to the MTJ with a diameter of 1.0 µm whose spin diode results are depicted in Fig. 2 of the main text.

Figures S2(a)-(b) show time traces of the simulated gyrotropic motion at a realistic approach (OFT + STT) for an in-plane field in the yy direction of 39.8 Oe. Similar to Figs. 3(d)-(e), an RF current density with a frequency of 81 MHz and an amplitude of 3 x 10^9^ A/m^2^ is applied. The difference relies in Fig. S2(b), where a vortex is set with a negative polarity (P = -1), in contrast with Fig. S2(a) that presents some of the data already showed in Figs. 3(d)-(e) of the main text (P = +1). The polarity does not influence the gyrotropic motion and the consequent rectification, which proves that, in this context, the incapacity of the experimental INIT to control the polarity is not a drawback.

Figures S2(c)-(e) show time traces of the simulated gyrotropic motion for different oscillation-driven mechanisms, as defined in the main text. These results were obtained at a zero in-plane magnetic field. The input RF current density has a frequency of 69 MHz and an amplitude of 3 x 10^9^ A/m^2^. As referred in the main text, the contribution of the OFT for the gyrotropic oscillation is negligible (note that the ΔM_Y_ scale of the OFT time traces is different from the other two panels), being the oscillation mainly driven by the STT.


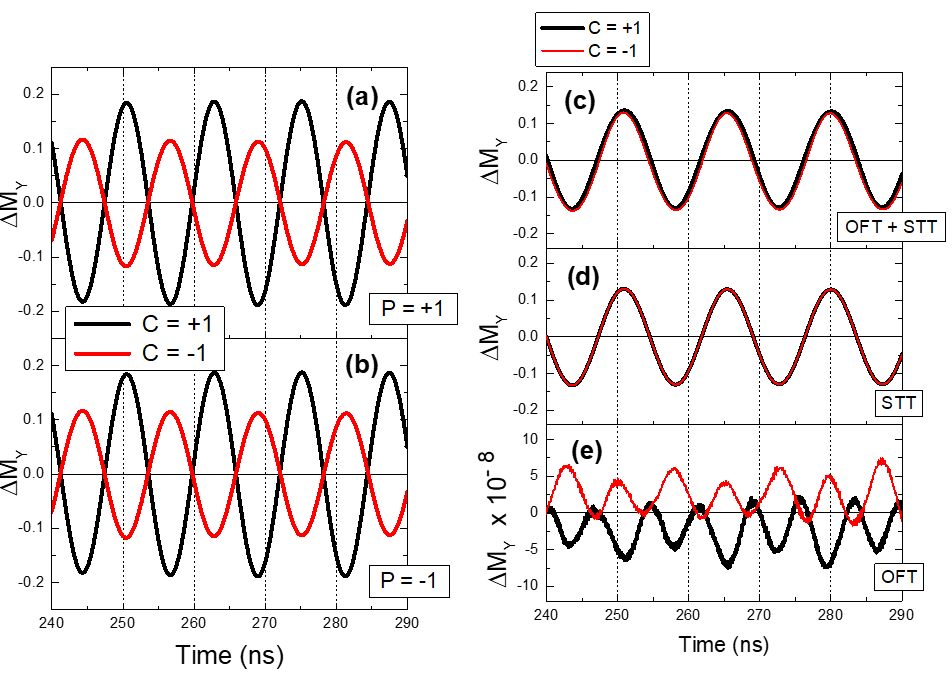


Figure S2: Simulated ΔM_Y_ time traces obtained for both chiralities at an in-plane field of 39.8 Oe, for (a) positive and (b) negative polarities. Both OFT and STT are considered. The results are obtained for a frequency of 81 MHz. (c) – (e) Simulated ΔM_Y_ time traces now obtained at zero in-plane field for different excitation mechanisms. The results are obtained for a frequency of 69 MHz. All time traces are obtained for an RF current density of 3.0 x 10^9^ A/m^2^.

Now, a full study of the spin diode evolution with the core displacement is presented. Figs. S3(a)-(b) show the module of the rectified voltage (V_REC_) obtained from simulations as a function of the in-plane field. These results are similar to the analysis depicted in Figs. 3(b)-(c) of the main text, but for different vortex core displacements. Also, as depicted in Fig. S3(c), V_REC_ is calculated as the peak-to-valley or valley-to-peak voltage. As expected, starting from an oscillation absence at zero field, there is a sustainable increase of V_REC_ with the field for the OFT-driven oscillation. For the same setpoint field, both chiralities show the same V_REC_ with symmetric spin diode curves [Figs. 3(b)-(c) of the main text], as a result of the 180° shift of the OFT-driven oscillation [Figs. 3(d)-(e) of the main text]. For the STT-driven oscillation, V_REC_ shows the same dependence for both chiralities, in line with the results shown in Figs. 3(d)-(e) of the main text, where the phase relation between ΔM_Y_ and J_RF_ (i.e. spin diode curve) does not change with the chirality. The results become quite interesting when both OFT and STT are considered. For C = +1, V_REC_ increases continuously, as a result of the phase relation between the OFT-driven ΔM_Y_ and J_RF_. For C = -1, one may define two main regions of the field domain. In the first region (H < 15 Oe), there is a continuous decrease of V_REC_ before reaching a zero oscillation setpoint. Now that the OFT-driven ΔM_Y_ shifts 180°, the increase of the OFT contribution with the field damps the oscillation to a smaller orbit, in this way decreasing V_REC_. This behaviour leads to a field setpoint where a perfect balance between the OFT and STT contributions stops the gyrotropic oscillation. In the same region, before reaching the absence oscillation setpoint, the STT contribution is still dominant over the OFT, meaning that, as depicted in Fig. S3(c), the spin diode curves for both chiralities are still similar, only with different amplitudes (both with a peak-to-valley lineshape). In the second region (H ≥ 15 Oe), the OFT becomes the dominant resonance mechanism, so that V_REC_ starts to increase and the lineshape of the spin diode curve for C = -1 is inverted [valley-to-peak lineshape in Fig. S3(d)].


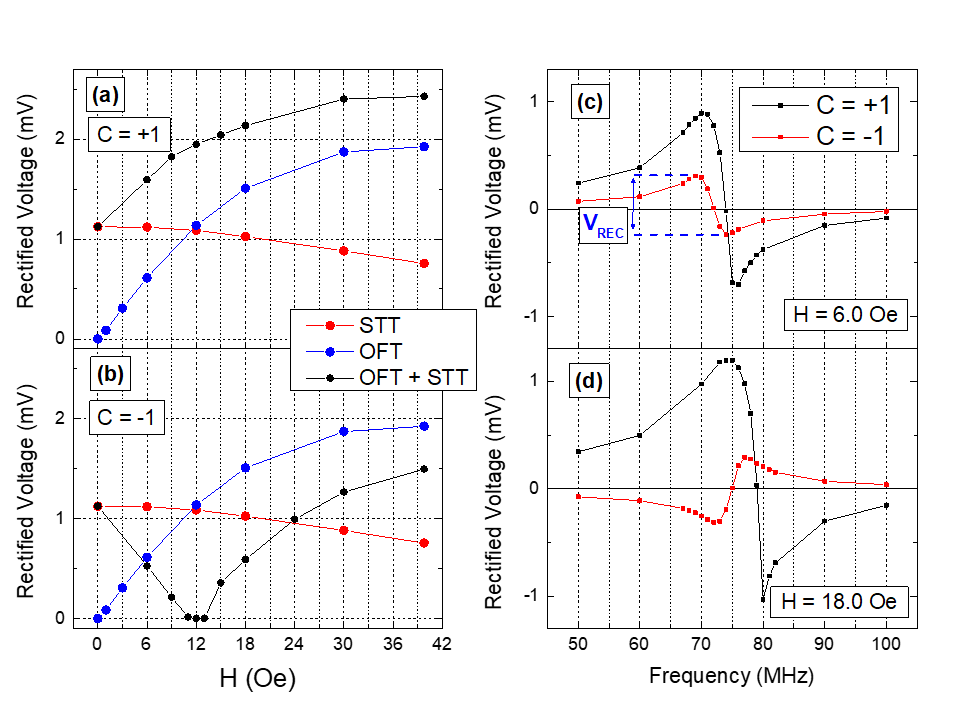


Figure S3: (a) Simulated rectified voltage calculated as a function of the in-plane magnetic field applied in the yy direction for the two different vortex chiralities. Again, the simulated spin diode measurements are presented for different excitation mechanisms: spin-transfer torque (STT), Oersted field torque (OFT) and both (OFT + STT). The results were obtained for an RF current density of 2.8 x 10^9^ A/m^2^. The simulated spin diode outputs are presented for a setpoint field of (c) 6.0 Oe and (d) 18.0 Oe. For the later, both OFT and STT are considered. The calculation of V_REC_ is explained in (c) as the module of the peak-to-valley voltage.
